# Epidemiology of Legionnaires’ Disease in Italy, 2004–2019: A Summary of Available Evidence

**DOI:** 10.3390/microorganisms9112180

**Published:** 2021-10-20

**Authors:** Matteo Riccò, Simona Peruzzi, Silvia Ranzieri, Pasquale Gianluca Giuri

**Affiliations:** 1 Servizio di Prevenzione e Sicurezza Negli Ambienti di Lavoro (SPSAL), AUSL—IRCCS di Reggio Emilia, Via Amendola n.2, I-42022 Reggio Emilia, Italy; 2 Laboratorio Analisi Chimico Cliniche e Microbiologiche, Ospedale Civile di Guastalla, AUSL—IRCCS di Reggio Emilia, I-42016 Guastalla, Italy; simona.peruzzi@ausl.re.it; 3 Department of Medicine and Surgery, School of Occupational Medicine, University of Parma, Via Gramsci n.14, I-43123 Parma, Italy; silvia.ranzieri@unipr.it; 4 Dipartimento Internistico Interaziendale, Struttura Operativa Semplice Dipartimentale “Medicina Infettivologica”, AUSL—IRCCS di Reggio Emilia, Ospedale “Sant’Anna”, I-42035 Castelnovo ne’ Monti, Italy; pasqualegianluca.giuri@ausl.re.it

**Keywords:** *Legionella* pneumophila typing, Legionnaires’ disease, cultivation, diagnosis, epidemiology

## Abstract

Legionnaires’ disease (LD) incidence has been increasing in several European countries since 2011. Currently, Italy is experiencing high notification rates for LD, whose cause still remains scarcely understood. We sought to summarize the available evidence on the epidemiology of LD in Italy (2004–2019), characterizing the risk of LD by region, sex, age group, and settings of the case (i.e., community, healthcare, or travel-associated cases). Environmental factors (e.g., average air temperatures and relative humidity) were also included in a Poisson regression model in order to assess their potential role on the annual incidence of new LD cases. National surveillance data included a total of 23,554 LD cases occurring between 2004 and 2019 (70.4% of them were of male gender, 94.1% were aged 40 years and older), with age-adjusted incidence rates increasing from 1.053 cases per 100,000 in 2004 to 4.559 per 100,000 in 2019. The majority of incident cases came from northern Italy (43.2% from northwestern Italy, 25.6% from northeastern Italy). Of these, 5.9% were healthcare-related, and 21.1% were travel-associated. A case-fatality ratio of 5.2% was calculated for the whole of the assessed timeframe, with a pooled estimate for mortality of 0.122 events per 100,000 population per year. Poisson regression analysis was associated with conflicting results, as any increase in average air temperature resulted in reduced risk for LD cases (Incidence Rate Ratio [IRR] 0.807, 95% Confidence Interval [95% CI] 0.744–0.874), while higher annual income in older individuals was associated with an increased IRR (1.238, 95% CI 1.134–1.351). The relative differences in incidence between Italian regions could not be explained by demographic factors (i.e., age and sex distribution of the population), and also a critical reappraisal of environmental factors failed to substantiate both the varying incidence across the country and the decennial trend we were able to identify.

## 1. Introduction

Legionellosis is a collective term encompassing two distinctive clinical syndromes caused by Gram-negative bacilli from the genus *Legionella*, i.e., Legionnaires’ Disease (LD) and Pontiac Fever (PF) [1,2,3]. Both disorders result if the bacteria are inhaled or aspirated but have strikingly different clinical characteristics. On the one hand, LD is a severe, sometimes fatal interstitial pneumonia, that shares several clinical features with pneumococcal and other bacterial pneumonia, with characteristic common extrapulmonary manifestations (i.e., renal failure, encephalopathy, pericarditis). On the other hand, PF is afebrile and generally flu-like illness persisting from two to six days, usually self-limiting and deprived of pulmonary parenchymal disease [2,3].

To date, 60 species of *Legionella* spp., and more than 70 distinct serogroups, have been identified [2,3], but the majority of human cases in Europe and North America is associated with *Legionella pneumophila* serogroup 1 (Lp1), the one originally described in the first documented outbreak in Pontiac, 1976 [4]. Non-Lp1 serogroups have been recently described as infrequent causes of hospital-related outbreaks of LD in Europe and North America [1,2,3,5], while *L. longbeachae* and *L. bozemanii* nowadays cause over half of the cases in Australia and New Zealand and increasing numbers in Scotland [6].

From a microbiological point of view, species of the genus *Legionella* have been reported worldwide, being nearly ubiquitously found in natural and artificial aquatic environments, where they colonize their natural hosts, i.e., amoeba and protozoa, mainly from genus *Naegleria* and *Acanthamoeba* [3]. To date, there is no evidence of zoonotic transmission, and also interhuman transmission has rarely occurred [1]. Therefore, it can be presumed that natural and artificial aquatic environments, as well as natural soil and potting soil/compost, are the only sources of human infections [7,8], with susceptible individuals becoming infected through inhalation of aerosols or aspiration of contaminated water from engineered products [9,10,11,12,13].

Exposure to *Legionella* elicits a strong immune response and the production of specific antibodies that last from several months up to 10 years after exposure [1,2,3]. Known risk factors for LD can be dichotomized into host-related and environmental ones. Host-related factors include: older age (>50 years), male sex, smoking history, chronic lung disease, diabetes, and various conditions associated with immunodeficiency (e.g., transplantation and/or chemotherapy) [2,3,8,14,15,16]. For instance, in the recent report from New York state, of the 1449 cases of LD, 88.2% had at least one underlying medical condition known as a risk factor for legionellosis [17]. Environmental risk factors associated with outbreaks of LD are travel, residence in a healthcare facility, and proximity to cooling towers, whirlpool spas, fountains [17].

However, according to the European Centre for Disease Prevention and Control (ECDC), in the large majority of European cases (approximately 70%), no specific environmental risk factors are actually identified in the 10 days before the onset of the clinical syndrome, thus suggesting a community-acquired infection. As a consequence, incident cases of LD are usually classified as community-acquired (CALD), travel-associated (TALD), and healthcare-associated (HALD). HALD are particularly relevant for both their crude incidence and their higher mortality, as both issues are influenced by the patients’ pre-existing pathologies and their level of immunocompetence [1,2,3,14,15,16].

Epidemiological studies about *Legionella* have reported a remarkable geographic variability in the overall prevalence of legionellosis, and particularly of LD [1,3,12], which could possibly be explained by the choice of different diagnostic tests (PCR, culture, or urine antigen test), efficiency of the surveillance systems, climate, and/or geographically linked risk factors [18,19,20]. However, there is some evidence that the notification rate for LD has increased both in North America and European Union/European Economic Area (EU/EEA), with both regions showing comparable notification rates and similar settings and epidemiology [21]. For example, the notification rate for UE/EEA increased between 2011 and 2018 from 1.2 to 1.8 cases per 100,000 inhabitants [18], while the estimated incidence in the city of New York saw an increase of 230% from 2002 to 2009 [17]. In the same decade, the annual age-adjusted mortality rate for LD in USA slightly increased from 0.038 per 100,000 population (95% CI 0.031 to 0.046) in 2000 to 0.040 per 100,000 population (95% CI 0.033 to 0.047) in 2010 [22].

The underlying drivers of this decennial trend are unknown, but possibly include higher testing rates, climate change with increasing occurrence of warm and wet weather conditions that facilitate the growth and spread of bacteria that in turn feed the proliferation of the hosts of *Legionella*, and the demographic transition, with more high-risk subjects belonging to an increasingly old general population [18,19,20].

However, it must be stressed that our awareness of the actual incidence of LD remains largely unknown, for several reasons. Firstly, most of our understanding comes from symptomatic patients, who present with CALD/TALD or HALD. As LD cannot be clinically or radiologically distinguished from pneumonia cases of different etiology, a low suspicion index means that a significant share of all diagnoses is simply missed because specific diagnostic testing is not performed [6,23,24]. As several studies have shown antibody levels in healthy populations ranging from less than 1 to 45.1% [1], the overwhelming majority of all infections usually occur unnoticed, with only a small fraction of all cases developing either PF or LD. Second, when testing is done in acute disease, clinicians mostly rely on urinary antigen tests (UAT), that represents 82 to 97% of diagnosis in Europe and in the United States, respectively [6,24]. UAT can be performed rapidly and with very high specificity for Lp1 but has a very low sensitivity for non-Lp1 antigens [3,8,25]. Alternative diagnostic tests and laboratory procedures (i.e., culturing, identification of the bacterium using paired serology, detection of the bacterium in tissue or body fluids by immunofluorescent microscopy, and genotypic polymerase chain methods), are in turn affected by specific shortcomings including higher costs and increased turnaround times, being therefore inconsistently deployed because of economic and/or practical reasons, and suggesting that a large share of all cases may remain simply undiagnosed.

In other words, LD is an important public health problem in terms of morbidity and mortality, particularly in certain high-risk groups, but our incomplete understanding of its epidemiology impairs an appropriate implementation of response measures, including environmental interventions, preventive campaigns focusing on the safety of potential sources of the infection, and risk communication. As a consequence, an updated and comprehensive reappraisal of surveillance data may be particularly useful to national and local health authorities.

In Italy, epidemiological surveillance for LD started in 1983, when the Legionellosis National Registry was established and managed by the Italian National Institute of Health (Istituto Superiore di Sanità, ISS). Notification of LD became mandatory in 1990 [8], and only in 2000, the Italian Institute of Health (Istituto Superiore di Sanità, ISS) issued its first guidelines for the prevention and control of legionellosis. This document was followed in 2005 by instructions targeted to microbiology laboratories, environmental control, tourist accommodation, and spas. Later, in 2015, all national recommendations, including those for hospitals, were included in a single updated document [8,26].

Even though earlier data suggest a significant increase in notification rates, an updated and comprehensive review of Italian data has not been reported [8,24,27,28]. Our aim was therefore to collect available evidence on the temporal and spatial patterns of LD in Italy, assessing the characteristics of incident cases in the subsequent timeframe of 2011–2019. Eventually, we performed a comprehensive analysis on the possible influence of environmental factors on the epidemiology of LD, focusing on climate and demographics factors.

## 2. Materials and Methods

### 2.1. Settings

Italy is administratively divided into 19 ordinary regions and 2 autonomous provinces (i.e., AP of Trento and Bolzano). With a surface of 301,340 km^2^ (116,350 sq mi), a total population of approximately 60 million inhabitants, and a population density of 201 people per square kilometer (520/sq. mi.), Italy is a densely but unevenly populated country in southern Europe. In fact, northwestern (Piedmont, Aosta Valley, Liguria, Lombardy) and northeastern regions (Veneto, AP of Trento and Bolzano, Friuli Venezia Giulia, and Emilia Romagna) encompass around 40% of the total area, but more than 45% of the total population, with low unemployment rates and high GDP per capita. Central (i.e., Lazio, Tuscany, Umbria, and Marche; 19% of total area), southern (i.e., Abruzzo, Apulia, Basilicata, Calabria, Campania, Molise), and Main Islands (i.e., Sicilia and Sardinia) are characterized by lower population density, lower economic development and higher unemployment rates that, in the past decades, were determinative of lower living standards for the inhabitants of southern regions, causing an intensive internal migration towards northeastern and northwestern Italy.

After nearly two decades characterized by stagnating population growth, in the time period 2001–2011, almost three million more Italians were registered, nearly all being young immigrants who therefore contributed to slow down the aging of the Italian population. According to available figures, people aged 65 and over increased steadily during past decades, now representing approximately 20% of the total population. Around 3% of them are assisted in the three main different kinds of Italian residential/institutional services: nursing homes, protected homes, and social health residential structures (i.e., structures focusing on older people or people with disabilities who require special care and support of medical, nursing and/or rehabilitation services).

### 2.2. Data Collection

Since the late 1990s, the national surveillance plan for *Legionella* infections implemented the publication of annual reports [8], available at: https://www.epicentro.iss.it/legionellosi/documentazione-italia, (accessed on 1 September 2021).

According to the present EU case definition of LD, and to the national guidelines for *Legionella* spp. control and prevention [13,24,26,29], a confirmed case of LD is a patient presenting clinical and/or radiological signs of pneumonia, with at least one of three laboratory criteria including: isolation of *Legionella* spp. from respiratory secretions or any normally sterile site; detection of Lp1 antigen in UAT; rise in specific antibody level to Lp1 in paired serum samples. A probable case is a patient presenting clinical and/or radiological signs of pneumonia associated with a single high level of specific antibodies to Lp1 (≥1:256), or a positive direct immunofluorescence test, or a positive PCR. Until 2012, cases were reported according to the guidelines of the European Working group for *Legionella* Infections (EWGLI) [30], with confirmed cases including subjects having a clinical or radiological diagnosis of pneumonia with laboratory evidence of one of the followings: isolation of *Legionella* species from clinical specimens, a positive UAT for Lp1 using validated reagents or kits, seroconversion (i.e., 4-fold or greater increase in titre) at indirect immunofluorescent antibody test for Lp1. A presumptive case was defined by either a clinical or radiological diagnosis of pneumonia with laboratory evidence of: detection of *Legionella* spp. nucleic acid in a clinical specimen; a positive direct fluorescence on a clinical specimen using validated *L. pneumophila* monoclonal antibodies; a single high titre for Lp1 antigen (≥1:128); a 4-fold increase in antibodies against other *Legionella* spp. or non-Lp1 infections.

Annual reports on LD include:

- the total number of diagnosed cases during the calendar year by gender, age group, and region of origin;
- the total number of probable/presumptive vs. confirmed cases;
- share of HALD, defined as patients residing in a hospital or nursing home for the entire 10 days before onset;
- subset of cases occurring in nursing homes for the entire 10 days before onset (available for 2007–2019 only);
- total number of TALD (either Italian and foreigner having being infected in Italy), defined as cases linked to tourist recreational facilities and tourist turnout;
- share of CALD, i.e., cases where no specific risk factor was identified in the 10 days before the onset of clinical symptoms, and therefore considered sporadic/community acquired;
- pre-existing medical conditions;
- diagnostic procedures performed in notified cases (i.e., urinary antigenic testing; isolation of the pathogen, PCR analysis)

For each study year, data on the Italian population, including year income for general population and renters, were obtained at a regional level from the Italian National Statistical Institute (ISTAT; http://demo.istat.it/, (accessed on 1 September 2021)) [31].

Pooled year meteorological data were obtained for each regional capital from the competent Regional Environmental Protection agencies (in Italian, ARPA). The Italian ARPA are the Italian environmental agencies, one for each region of Italy (excluding Trentino-Alto Adige/Süd Tirol, which has been split for the two Autonomous Provinces of Trento and Bolzano). When more weather stations were available at the same geographical level for the same timeframe, mean values were calculated.

### 2.3. Statistical Analysis 

We performed a descriptive analysis of the surveillance data, i.e., geographical and temporal distribution of LD cases, with their respective demographic characteristics (age, sex), and clinical outcome, where available. Crude incidence rates (CIR) were calculated both at a national and regional level, with subsequent estimates of case fatality ratio (CFR), and crude mortality rates. Age-adjusted incidence rates (ASR) were calculated at a national level assuming the European standard population as reference [32]. In order to ascertain the impact of HALD over the total of cases locally reported, the share of HALD reported by the index region (i) was compared to the share of all HALD cases at a national level (I^HALD^ = i^HALD^/Total^HALD^), with a similar estimate for the whole of reported LD by the same region (I^LD^ = i^LD^/Total^LD^). The regional HALD Risk Ratio was then calculated as I^HALD^/I^LD^.

We analyzed then the corresponding annual incidence rates between 2004 and 2019, dichotomized by convenience in 2004 to 2011 vs. 2012 to 2019. Such cut-off was identified as, since 2012, European countries belonging to the European Legionnaires’ Disease Surveillance Network (ELSDNet), including Italy, in accordance with the 2012 EU/EEA case definition, reported only cases with acute pneumonia [8,30,33], allowing a more appropriate comparison with European data. Annual trends for Italy and other European Countries [34,35,36] were initially compared through the Pearson’s correlation test, and then assessed by normalizing the annual rates by the maximum number of new cases.

The relationship between the number of LD cases and both meteorological (i.e., average daily temperature, average precipitation rates) and demographic factors (i.e., share of population aged 50 years or more, annual income in the general population and in renters) was initially investigated through the Pearson’s correlation test. Incident Rate Ratios (IRR) with their correspondent 95% CI were calculated in a Poisson regression model that included as the outcome variable either the yearly incident rates for LD (assessed at regional level) and the healthcare-related cases. The explanatory variables were represented by meteorological and demographic factors. In the analyses, meteorological data of the regional capital were assigned to all LD cases that occurred in all municipalities of the province.

All calculations were performed on R 4.0.3 (R Core Team (2020). R: A language and environment for statistical computing. R Foundation for Statistical Computing, Vienna, Austria. URL https://www.R-project.org/, (accessed on 1 September 2021)) [37] by means of packages epiR (v. 2.0.19), EpiReport (v 1.0.1), fmsb (0.7.0), msm (1.6.8), sandwich (3.0-0).

### 2.4. Ethical Approval 

No ethical approval was needed for this study, as no individual data were identifiable, and since we analyzed and presented only aggregated data.

## 3. Results

### 3.1. Demographics

A total of 23,554 cases of LD were reported in Italy between 2004 and 2020 (Table 1), 66.4% of them occurring after 2012. Of them, 22,891 were confirmed ones (97.2%), and the share of probable/presumptive decreased from 4.6% in 2004–2011 to 1.9% in 2012–2019. The main diagnostic option was represented by UAT (95.3%), while only 3.2% of all cases were identified by serology, whose share decreased from 5.7% in 2004–2011 to 1.9% in 2012–2019 (*p* < 0.001).

The majority of cases (70.4%) occurred in males and in older age groups (i.e., 19.3% in 50 to 59 year-olds; 22.1% in 60 to 69 year-olds; 22.1% in 70 to 79 year-olds; and 18.4% in subjects aged 80 years or older). The occurrence of LD increased in all age groups, but the representation of age groups > 50 years significantly increased from 77.4% in 2004–2011 to 84.4% (*p* < 0.001). An underlying chronic disease was reported in 52.5% of cases.

A total of 17,193 cases (73.0%) were classified as CALD, as no specific risk factors were identified in the 10 days before the onset of symptoms, and their corresponding share increased from 65.8% in 2004–2011 to 76.6% in 2012–2019 (*p* < 0.001). On the contrary, HALD accounted for 1394 (5.9% of the total sample), and while their share substantially halved from 8.6% to 4.6% during the overall timeframe (*p* < 0.001), raw figures remained quite consistent (i.e., 84.4 cases/years 2004 to 2011, compared to 89.4 cases/years, t = −0.443 and *p* = 0.666 in Student’s *t*-test). Unfortunately, data on residential homes were available only for the time period 2007–2019, with 523 total diagnoses, i.e., 2.5% of 21,158 total cases diagnosed in the respective timeframe. Moreover, a total of 209 cases were linked to dental care (5.9%), with no significant differences between 2004–2011 and 2012–2019 (0.9% vs. 0.9%, *p* = 0.604), with 311 diagnoses associated with swimming pools. While the total number of notified cases decreased from 193 in 2004–2011 to 118 in 2012–2019, the respective share dropped from 2.4% to 0.8% (*p* < 0.001).

Eventually, 4967 cases were identified as TALD (21.1%), and again the corresponding share decreased from 25.6% in 2004–2011 to 18.8% in 2012–2019 (*p* < 0.001). Among TALD cases, 2188 occurred in foreigner travelers who presumptively were infected in Italy (9.3%), and 2779 cases in Italian travelers (11.8%). Of them, 257 (1.1%) were exposed to *Legionella* spp. abroad. Since 2013, involved structures increased from 47 to 81, and around one-third of them were previously characterized by incident cases (i.e., 30.3%, range 22.9% in 2019, 35.6% in 2013).

Unfortunately, reliable estimates for the nights spent in Italian Hotels and hospitality facilities for the assessed timeframe were not available, while a similar estimate was provided from the ISTAT for Italian international travelers. As a consequence, a crude incidence was calculated for this specific subgroup of travelers, with estimates ranging from 0.092 per 1,000,000 nights spent abroad in 2005 to 0.266 in 2018, and an average of 0.148 (95% CI 0.110–0.185) (Table A1).

As reported in Table 2, crude incidence rates for LD increased from 1.048 cases per 100,000 (95% CI 0.958–1.139) in 2004 to 5.343 per 100,000 (95% CI 5.131–5.555) in 2019. The average yearly incidence was 2.464 cases per 100,000 (95% CI 1.880–3.048). AIR showed the very same trend, increasing from 1.053 cases per 100,000 (95% CI 0.869–1.237) in 2004 to 4.669 per 100,000 (95% CI 4.251–5.088) in 2019 (pooled estimate: 2.285 cases per 100,000, 95% CI 1.798–2.772).

**Figure 1:**
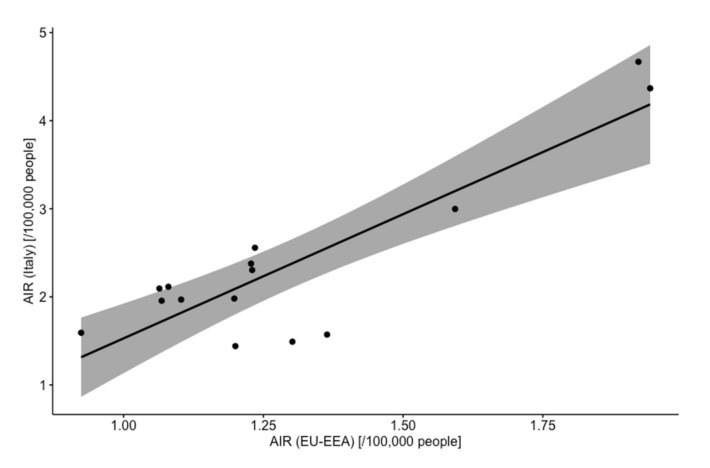
Correlation of annual Age-adjusted Incidence Rates (AIR) for Legionnaires’ disease in Italy and EU-EEA countries. As shown Italian and European estimates were well correlated (r = 0.871, 95% CI 0.647 to 0.956, *p* < 0.001).

AIR of Italy and EU-EEA countries were compared for the timeframe 2004–2019 and were well correlated (r = 0.871, 95% CI 0.647 to 0.956) (Figure 1).

**Figure A1:**
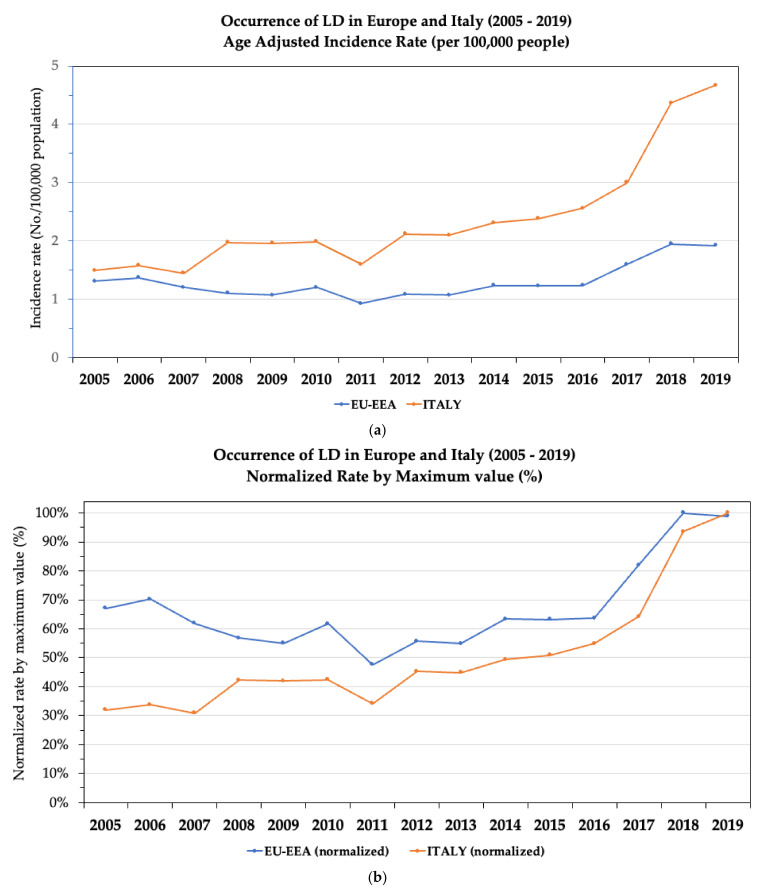
Comparison of Italian and European data for Legionnaires’ disease (LD) in the time period 2005–2019, as: Age Adjusted Incidence Rates (AIR) (**a**), AIR normalized by maximum value (**b**).

However, when the trend was assessed either as annual incidence estimates and incidence rates normalized by the maximum value of the assessed timeframe, a quite distinctive pattern was identified, with Italy exhibiting a surge of notified cases that substantially outmatched that of EU-EEA since 2012 (Figure A1).

### 3.2. Geographic Distribution

Focusing on the geographic origin of reported cases, the large majority them were from the northern region, i.e., 43.2% from northwestern Italy, and 25.6% from northeastern Italy. During the assessed timeframe, the share of cases reported from northeastern regions increased from 21.9% to 27.4%, while the corresponding proportion of cases from northwestern Italy decreased from 47.2% to 41.1% (*p* < 0.001).

As shown in Table 3, the regions characterized by the higher share of LD cases during the assessed timeframe were Lombardy (No. 7829, 33.3%) and Emilia Romagna (No. 3035, 12.9%), followed by Lazio (No. 2175, 9.2%), and Tuscany (No. 1994, 8.5%). However, as these areas are quite heterogeneous in terms of the reference population, the highest values of CIR were reported from the AP of Trento (i.e., 7.919 cases per 100,000; 95% CI 7.749–8.088), followed by the aforementioned regions of Lombardy (i.e., 4.999 cases per 100,000; 95% CI 4.953–5.046), and Emilia Romagna (i.e., 4.316 cases per 100,000; 95% CI 4.200–4.433). On the contrary, CIR well below 1 case per 100,000 was accounted for several regions from southern Italy and Major Islands, i.e., Puglia (0.670, 95% CI 0.626–0.714), Sardinia (0.470, 95% CI 0.407–0.553), Calabria (0.263, 95% CI 0.218–0.308), Sicily (0.225, 95% CI 0.206–0.244), and Molise (0.202, 95% CI 0.001–0.409).

Interestingly enough, AP of Trento was also characterized by the highest share of cases occurring as HALD (i.e., 18.0%), followed by Basilicata (16.2%), Calabria (9.7%), and Piemonte (9.1%), while in Lombardy and Emilia Romagna it was estimated to 7.2% and 6.3%, respectively.

The corresponding Risk Ratio was therefore 2.885 (95% CI 2.479–3.232) for the AP of Trento, followed by Lombardy (2.126, 95% CI 2.056–2.197), Aosta Valley (1.635, 95%CI 1.302–1.967), and Emilia Romagna (1.608, 95%CI 1.322–1.894).

Focusing on larger areas, CIR was higher in northwestern Italy (3.527, 95% CI 2.895–4.158), followed by northeastern Italy (2.572, 95%CI 1.885–3.258), and central Italy (2.190, 95% CI 1.757–2.622), and corresponding Risk Ratios were 1.697 (95%CI 1.500–1.894), 1.244 (1.055–1.432), and 1.068 (95% CI 0.951–1.185). In other words, while in northern regions the occurrence of LD among healthcare facilities and residential homes was substantially increased, the likelihood of new diagnoses in such settings in southern Italy (Risk Ratio 0.322, 95%CI 0.271–0.374), and in the Islands (0.117, 95% CI 0.091–0.143) was by contrast starkly reduced.

### 3.3. Deaths

**Figure 2:**
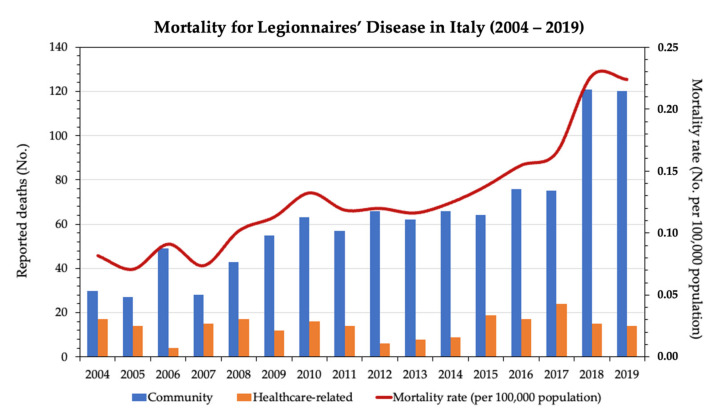
Mortality of Legionnaires’ Disease (LD) in Italy (2004–2019). Incident deaths are reported separately for community-occurring and healthcare-related cases.

A total of 1223 deaths were reported between 2004 and 2019. The absolute number of deaths increased from 47 in 2004 to 134 in 2019, with a crude mortality rate ranging from 0.082 per 100,000 population (95%CI 0.060 to 0.109) in 2004 to 0.224 (95%CI 0.188 to 0.265) in 2019, and a pooled estimate of 0.122 events per 100,000 population (95%CI 0.104 to 0.143) (Figure 2).

CFR, including both CALD and HALD, was estimated to be 5.2% for the time period 2004–2019. As shown in Table 4, the majority of deaths occurred in non-healthcare settings cases (1002 vs. 221 HALD), with respective CFRs of 4.6% and 11.5% for CALD and HALD, respectively (*p* < 0.001). When national data were broken down by the timeframe (i.e., 2004–2011 vs. 2012–2019), CFR decreased from 5.8% to 4.9%, with a similar trend in non-healthcare (i.e., 5.0% vs. 4.5%), and in healthcare-related cases (13.3% vs. 10.2%).

### 3.4. Analysis of Risk Factors

Consistently with previous estimates, by assuming the timeframe 2004–2011 as the reference (Table 5), the subsequent time period 2012–2019 was associated with an increased RR for new diagnoses (1.936, 95% CI 1.884–1.989). The risk for incident LD increased in males compared to female individuals (RR 2.522, 95% CI 2.453–2.594), and in older age groups. By assuming the age group of <40-year-old as a reference, RR increased from 4.320 (95% CI 4.051–4.606) in the age group 40 to 49, to 6.259 (95% CI 5.894–6.647) in the age group 50 to 59, 8.145 (95% CI 7.676–8.462) among subjects 60- to 69-year-old, and peaked in the age group 70 to 79 years (RR 9.610, 95% CI 9.058–10.197), followed by ≥80-year-old subjects (RR 9.447, 95%CI 9.361–10.565).

Regarding the geographic origin of reported cases, by assuming northwestern Italy as a reference, a substantially reduced risk for new diagnoses was identified in all other areas, from northeastern Italy (RR 0.819, 95%CI 0.793–0.845), to central Italy (RR 0.643, 95%CI 0.622–0.665), and particularly in southern regions (RR 0.209, 95%CI 0.199–0.220), and major islands of Sicily and Sardinia (RR 0.071, 95%CI 0.064–0.080).

Eventually, when focusing on the LD-associated deaths, cases occurring in healthcare facilities and residential homes were associated with a nearly doubled risk for eventual death compared to CALD (RR 2.489, 95% CI 2.169–2.858), which was substantially decreased in 2012–2019 timeframe compared to 2003–2011 (RR 0.835, 95%CI 0.746–0.934).

When meteorological factors were taken into account alongside the share of the population aged 50 years or more, and the year income (both for renters and general population), the latter factors were significantly correlated with incidence rates for LD (r = 0.405, 95% CI 0.175–0.594, *p* < 0.001; r = 0.695, 95% CI 0.540–0.804, *p* < 0.001; r = 0.643, 95% CI 0.470–0.768, *p* < 0.001; respectively) (Table 6). Interestingly enough, the occurrence of HALD was in turn negatively correlated with air temperatures (r = −0.309, 95% CI −0.518 to −0.067), income of renters (r = −0.416, 95% CI −0.602 to −0.188, *p* < 0.001), and income of the general population (r = −0.259, 95% CI −0.476 to 0.012, *p* = 0.041).

In fact, when climate data were included in a Poisson regression model with the share of older residents over the total population (i.e., aged 50 years or more), air temperatures, daily precipitation rates, and annual income (both for general population and renters), a more distinctive pattern was identified. As shown in Table 7, a decreased estimate for the average air temperature was identified for both incident cases as a whole, and HALD cases (+1.0 °C; IRR 0.807, 95%CI 0.744–0.874 and IRR 0.884, 95% CI 0.783–0.999, respectively). On the contrary, while year income of renter (+1000 €/year) was associated with an increased occurrence of LD cases (IRR 1.238, 95% CI 1.134–1.351), it was characterized as a negative explanatory variable for healthcare-related cases (IRR 0.888, 95% CI 0.816–0.966).

## 4. Discussion

Our review of surveillance data suggests that the notification rates of LD in Italy have substantially increased across the assessed timeframe 2004–2019. In other words, not only do our analyses mainly confirm the surge of LD cases reported by Rota et al., since 2013 [8], but our estimates are also quite consistent with available European data [34,35,36]. More precisely, the pooled incidence rate has increased 4.5 times since 2003, with an AIR climbing from 1.053 per 100,000 in 2003 (95% CI 0.869 to 1.237) to 4.669 per 100,000 (95% CI 4.251 to 5.088) in 2019. In fact, during the last decade, not only has Italy consistently reported the highest raw number of LD in all of western Europe, but the incidence rates have increased over time, being second only to Slovenian figures (i.e., 5.1 per 100,000 in 2015; 4.5 per 100,000 in 2016; 5.7 per 100,000 in 2017; 7.7 per 100,000 in 2018; 9.4 per 100,000 in 2019) [20,34,35,36,38]. Notably, when Italian AIRs were compared to available EU-EEA estimates for 2005–2019, the corresponding ratio regularly increased from 1.1 in 2005 to 2.4 in 2019 (average of 1.8 for the whole timeframe, but 1.5 for 2005–2011 and 2.1 for 2012–2019). Collectively, such results point towards an increased risk for LD in Italy compared to most European countries, and particularly in the last decade.

To date, this upsurge is a common and not well-understood feature of nearly all high-income countries. Several explanations have been proposed [3,8,20,34,35], but none of them is either satisfying nor explicative [2,3,9,10,38]. In fact, we are unable to rule out that this increase in case numbers could be nothing more than an artifact. Better awareness of medical professionals towards a respiratory disorder otherwise deprived of clinical diagnostic features, associated with improved detection systems and changes in clinical diagnostic methods may have led to the increased testing frequency in high-risk individuals with clinical signs of pneumonia, particularly through UAT, and hence to an increase in detected cases [8,17,24,26,27,28]. In other words, we cannot rule out that our data could have been affected by a “diagnostic epidemic” too, rather than representing an actual epidemic of the pathogen. As a corollary, we could also speculate that the lack of appropriate testing in the previous decades could have impaired our understanding of the true LD burden of disease, which is progressively ascertained [17,39,40]. However, this explanation is quite unlikely. Not only were urinary diagnostic tests introduced more than 20 years ago [41], well before the surge in LD notification rates (around 2013), but the proportion of positive cases on the whole of collected specimens remained quite consistent, and also the share of LD diagnosed by means of urinary assays has been substantially stable in the last years [1,41]. Moreover, it should be kept in mind that UAT, despite its convenience and the relatively reduced turnaround times when compared to alternative diagnostic methods (i.e., culturing, serology, immunofluorescent microscopy, and genotypic polymerase chain reaction) [1], is reliable only for Lp1 strains [3,10,21,25]. As 95.3% (and 96.8% since 2012) of reported cases were detected through UAT, we cannot rule out that official estimates might be somewhat underestimating the actual burden of disease.

Another possible explanation has been found in the ongoing demographic transition. Since the earlier reports, older age has been identified as the main risk factor for LD, and also in our data people aged 40 to 49 years old had an RR of developing LD that was 4.320 (95% CI 4.051 to 4.606) higher than in people younger than 40 years old, and it progressively increased to 6.259 (95% CI 5.894 to 6.647) in the age group 50 to 59 year-olds, 8.145 (95% CI 7.676 to 8.462) in the age group 60 to 69, 9.610 (95% CI 9.058 to 10.197) among individuals aged 70 to 79 years, and 9.447 (95% CI 9.361 to 10.565) in older subjects. In other words, the steady increase in the age of European, and particularly of Italian populations, has progressively overstretched the share of individuals being at high risk to develop LD, then contributing to the overall surge of incident cases. However, even though the proportion of subjects aged 50 years or more in the general Italian population has increased from 38.7% in 2004–2011 to 42.5% in 2012–2019, the occurrence of LD in these age groups has increased even more steadily (i.e., 77.4% in 2004–2011 compared to 84.4% in 2012–2019). Moreover, the raw number of individuals reporting pre-existing chronic diseases nearly doubled between 2004–2011 and 2012–2019 (i.e., 4564 vs. 7800), but the corresponding share of total cases decreased from 57.7% in 2004–2011 to 49.8% in 2012–2019. In other words, demographics represent a valuable explanation of the decennial trend, both at the Italian and European level, but it is hardly the only one.

More recently, climate change and several environmental features have been advocated as a possible contributor to the upsurge of notification rates for LD and other pathogens. This is particularly intriguing as Italy, and particularly its northern regions, has been severely hit by climate change in past decades [42,43,44,45,46,47], with well-documented consequences on the ecology of several infectious diseases [48,49]. Although *Legionella* spp. is common in the environment, dry environments do not support them, and the pathogen is particularly sensitive to drying conditions [50]. Even though available source data lacked the monthly occurrence of reported cases, and we were therefore unable to perform an accurate analysis of the seasonality of LD in Italy, a striking and well-known epidemiologic feature of LD, and particularly of CALD, is seasonality, as more cases are reported during the summer. Some evidence indicates that high environmental temperatures (and more precisely, large temperature excursions), and high relative humidity during the warm season, together with increased rainfall during the late spring months, would drive the summer spike in incidence [51,52,53]. As hinted by Hicks et al., it is reasonable that heavy rain can both favor the proliferation of host microorganisms or increase organic sediments in the water network which, in turn, support the growth of *Legionella*.

Still, multivariable analysis by means of a Poisson regression model identified air temperature as a negative effector (IRR 0.807, 95% CI 0.744 to 0.874), while precipitation rate was actually unrelated to the notification rate. In other words, our estimates were hardly comparable with available evidence but, contradictory as they were, they may find several explanations.

To begin with, it should be stressed that our analyses were forcibly coarse. Retrieved data were based on the region of origin: indeed, Italian regions represent a secondary administrative level, including quite large and heterogeneous areas. The reliability of the multivariable analysis may thus have been affected by other factors, including the economic development of assessed areas, and the appropriate notification of incident cases. For instance, being central and southern regions of Italy characterized by a warmer climate and lower economic development, the characterization of environmental factors and the annual income of renters as a potential risk factor (IRR 1.238, 95%CI 1.134 to 1.351) should be cautiously assessed.

In fact, a diffuse under-reporting in central and southern regions had already been addressed [8] and advocated as an explanation for the extensive heterogeneity among prevalence data we retrieved. In other words, the geographic trend in notification rates we identified, with estimates significantly decreasing from northwestern regions to northeastern, central and southern regions, and lower rates in Sicily and Sardinia, rather than stressing the relevance of environmental factors could be nothing more than an artifact.

The potential impact of misreporting on overall estimates is also hinted at by analysis of HALD cases. On the one hand, HALD cases are usually unrelated to environmental features; in fact, despite the substantial reduction of their proportion on all reported cases from 2004 (15.9%) to 2019 (4.5%), the raw number of actual reported cases remained quite consistent across the study period (84.4 cases/years 2004 to 2011, compared vs. 89.4 cases/years). On the other hand, calculation of HALD Risk Ratio mirrors the estimates for the overall incidence rates, suggesting that mostly in northern regions (i.e., 1.687, 95% CI 1.500 to 1.894 for northeastern regions, 1.244, 95% CI 1.055 to 1.432 for northwestern regions) their occurrence has been disproportionally high—or, conversely, that in most the central Italy, in southern Italy, as well as in Sicily and Sardinia, the occurrence of HALD cases was disproportionally low during the assessed timeframe.

Interestingly, official data on the consumption of antimicrobial drugs suggest a mixture of misreporting and heterogeneous management of pneumonia cases as another reliable explanation for retrieved data. According to the Italian Medicines Agency (i.e., Agenzia Italiana del Farmaco, AIFA in Italian), during the last decade, northern regions have experienced a sustained decrease in daily consumption of antimicrobial drugs having intracellular activity such as Fluoroquinolones (2.5 defined daily doses [DDD]/1000 persons in 2017 compared to 1.6 in 2019). To date, central (3.3 DDD per 1000 persons in 2017, and 2.2 DDD per 1000 persons in 2019) and southern regions (4.0 DDD per 1000 persons in 2017, and 2.9 DDD per 1000 persons in 2019) still report higher consumption rates (see Table A2) [54,55]. As clinical features of LD are quite indistinctive, it is reasonable that a large share of cases occurring in central and southern Italy are still treated with broad-spectrum antimicrobial drugs: as *Legionella* spp. remains quite sensitive to Fluoroquinolones, without early microbiological testing, the clinical recovery may hinder a proper diagnosis, with consequent under-reporting [9,10,11]. On the contrary, where guidelines have promoted the use of drugs such as macrolides and penicillin, less effective against *Legionella* spp., clinicians may more frequently perform diagnostic testing in order to identify the pathogen, properly tailoring the antimicrobial treatment, but also leading to an increased number of diagnosed cases of LD [56,57]. As current Italian guidelines do recommend the systematic referral to diagnostic tests for community-acquired pneumonia, including the UAT, the increasing share of CALD may be otherwise explained through an increased sensitivity of the clinicians towards LD.

Notwithstanding, climate change and economic development may have influenced the increasing occurrence of LD in a more subtle way, i.e., through the ever-increasing spread of air-conditioning systems, particularly in the community, in order to cope with the rising summer temperatures, and the progressive shift from heating systems based on steam and hot water to heat pumps. Air-conditioning systems and convector radiators are (alongside drinking fountains, hot tubs, sinks, toilets, sprinklers, and showers) well understood as common sources of *Legionella*. In fact, the pathogen tends to flourish when the facilities are not maintained in proper terms, e.g., water is sufficiently warm to allow the proliferation of *Legionella*, stagnant, deprived of chemical disinfectants, and/or pipes are progressively corroded, with a resulting abundance of nutrients. Moreover, Europe, and most prominently Italy, is not only an aging continent—but also its urban infrastructures are aging as well. In large part, the water distribution system is very antiquated; as water travels through the distribution system and enters a building, it can lose disinfectant as well as interact with the materials, temperatures, and design of the building’s plumbing, with subsequent risk of its contamination by *Legionella* spp., that are then dispersed in the environment.

The increasing use of air-conditioning systems in a rapidly aging population may also explain the increasing occurrence of CALD compared to HALD. Focusing on our data, while in 2004 HALD cases accounted for 15.9% (95% CI 13.5 to 18.3), the corresponding share decreased to 4.5% (95% CI 4.4 to 4.8) in 2019, with a nadir of 4.0% (95% CI 3.5 to 4.5) in 2018. In fact, Italian health authorities have progressively implemented a series of preventive measures to avoid HALD cases, including HEPA (High-Efficiency Particulate Air) filters, that show only a limited pairing on the legal framework for domestic plants. An indirect confirmation may be found through the analysis of the corresponding trend in TALD.

TALD cases have played a central role in LD history since the first description of this disorder, and travel is a well-known risk factor for LD for a variety of reasons. First, water systems of accommodation sites are often complex, with a large number of outlets (e.g., showers) [58]. Therefore, not only are water systems of accommodation sites sometimes not regularly flushed, with water stagnation that favors the growth of *Legionella*, but the length of pipe systems impairs an adequate control of water temperatures. Moreover, accommodation sites are likely to have facilities such as swimming pools and whirlpool spas, which are in turn associated with an increased risk of LD and that, particularly in seasonal accommodation structures, are not periodically managed and sanitized [58]. Last but not least, hotels may host a large number of visitors, who might be exposed to the same source during their stay, with resulting clusters and even outbreaks [30,53,59]. As a consequence, specifically targeted interventions have been promoted not only at the Italian level, but more broadly at the European level, including the implementation of an integrated surveillance network in all EU-EEA countries [30,34,38]. Not coincidentally, even though the number of national and international tourists and tourism-related facilities has more than doubled during the last decade (according to the estimates of ISTAT, the number of international travelers in Italy skyrocketed from 165 million in 2010 to nearly 400 million in 2019), the raw number of notified cases between 2004–2011 and 2012–2019 exhibited a far more limited increase, from 2024 to 2943, with a substantial decrease for the proportion of TALD on the total of LD (i.e., 25.6 to 18.8%). It is, therefore, reasonable to think that the aimed interventions of health authorities on water and climatization systems of accommodation sites may have been instrumental in limiting the increase of new TALD cases.

The possible relevance of factors other than demographic and environmental ones are also suggested by the small but significant difference that was appreciated across the assessed timeframe regarding the gender of reported cases. Individuals of male gender were consistently more often affected than females (RR 2.522, 95% CI 2.453 to 2.594), but they accounted for 71.7% of total cases in 2004–2011 compared to 69.7% in 2012–2019. The higher occurrence of LD in males compared to females has been repetitively but unsatisfying inquired [8,17]. While it is quite unlikely that differences in health-seeking behavior may have resulted in such differences [17], other factors such as smoking habits and occupational exposures hardly explain both the higher risk of males and the progressively increasing share of female cases [60,61].

*Limitations*. Albeit interesting, our data are affected by significant shortcomings. First, we have drawn our estimates from National Bulletins, and therefore we were able to summarize and analyze only information preventively reported by National Authorities. As a consequence, not only do we lack significant data about the demographics of LD cases, with resulting uncertainties in eventual estimates, but significant information about the reported signs and symptoms were not available to our analysis. This is particularly interesting, as an accurate analysis of clinical features of reported cases may either confirm or dismiss the hypothesis that the upsurge of LD cases should be more properly retained as an intensification of testing for *Legionella* [2,3,25,38,62]. Second, National Bulletins obviously included reported cases, but both previous remarks and the significant heterogeneity in incidence estimates hint for an inconsistent and often inappropriate notification of incident cases to the competent health authorities [8,27,39,40]. Such heterogeneities, coupled with the potential unreliability of some source data necessarily recommend a cautious generalization of our results. Third, we are totally deprived of information about the seasonality of reported cases. As a consequence, an appropriate analysis of environmental factors—including climate factors such as air temperature, and humidity, but also the availabilities of appropriate facilities (i.e., air-conditioning, thermal convectors, etc.), is to date either difficult or substantially biased from its roots [50,62]. Eventually, it must be stressed that our estimates only included LD, while the occurrence of PF was not ascertained as it is not included in the current case definition for LD by the European Union [38].

## 5. Conclusions

In conclusion, the present summary of Italian national reports on LD suggests that the annual notification rates of LD have substantially increased across the assessed timeframe. Available estimates hint for substantially higher notification rates in northern regions (mostly, highly developed regions from northwestern Italy) compared to southern ones and main islands. Interestingly, the upsurge in new LD cases mainly affected CALD and TALD, while HALD remained quite consistent in terms of raw number, being more frequently reported from northern regions than from southern ones. At the moment, no comprehensive explanation may be inferred, rather suggesting the interaction of several different factors. Even though most of the aforementioned epidemiological features appear very similar to those observed in other European countries and in the USA as well, suggesting a significant role for demographic transition and climate change (the latter both directly and indirectly), quite heterogeneous regional policies in terms of the testing of individuals with clinical signs and symptoms of pneumonia, and the suspected under-reporting of LD cases to competent health authorities may have contributed to the conflicting epidemiological features we have collected and reported. Therefore, in regions with persistent low notification rates, ad hoc studies should be designed and performed in order to assess reasons for under-ascertainment.

## Figures and Tables

**Table 1:** Summary of collected data about Legionnaires’ Disease (LD) cases in Italy (2004–2019). Notes: until 2012, LD cases were reported as confirmed vs. presumptive according to the EWGLI guidelines, then as confirmed vs. probable according to the EU guidelines. CALD = community-acquired LD; TALD = travel-associated LD; HALD = healthcare-associated LD.

	Total (No./23,554, %)	2004–2011 (No./7904, %)	2012–2019 (No./15,650, %)	*p* Value (Chi Squared Test)
Case definition				<0.001
Confirmed Cases	22,891, 97.2%	7544, 95.4%	15,357, 90.1%	
Probable/Presumptive cases	653, 2.8%	360, 4.6%	293, 1.9%	
Diagnosis				<0.001
Isolation of the Pathogen	365, 1.5%	154, 1.9%	211, 1.3%	
Urinary Antigen	22,447, 95.3%	7302, 92.4%	15,145, 96.8%	
Serology	742, 3.2%	448, 5.7%	294, 1.9%	
Gender				<0.001
Males	16,572, 70.4%	5670, 71.7%	10,902, 69.7%	
Females	6975, 29.6%	2224, 28.3%	4741, 30.3%	
Unknown	7, <0.1%	7, <0.01%	0, -	
Age group				<0.001
<40	1384, 5.9%	649, 8.2%	735, 4.7%	
40 to 49	2839, 12.1%	1137, 14.4%	1702, 10.9%	
50 to 59	4555, 19.3%	1480, 18.7%	3075, 19.6%	
60 to 69	5194, 22.1%	1681, 21.3%	3513, 22.4%	
70 to 79	5217, 22.1%	1714, 21.7%	3503, 22.4%	
80 or greater	4334, 18.4%	1225, 15.5%	3109, 19.9%	
Unknown	31, 0.1%	18, 0.2%	13, 0.1%	
Chronic diseases	12,364, 52.5%	4564, 57.7%	7800, 49.8%	<0.001
Geographic Origin				<0.001
Northwestern Italy	10,153, 43.2%	3725, 47.2%	6428, 41.1%	
Northeastern Italy	6015, 25.6%	1725, 21.9%	4288, 27.4%	
Central Italy	5368, 22.8%	1819, 23.1%	3549, 22.7%	
Southern Italy	1712, 7.3%	536, 6.8%	1176, 7.5%	
Islands	306, 1.3%	97, 1.2%	209, 1.3%	
CALD	17,193, 73.0%	5201, 65.8%	11,992, 76.6%	<0.001
HALD	1394, 5.9%	679, 8.6%	715, 4.6%	<0.001
Residential homes *	523, 2.5%	142, 2.6%	381, 2.4%	0.589
Dental care	209, 0.9%	74, 0.9%	135, 0.9%	0.604
Swimming pool	311, 1.3%	193, 2.4%	118, 0.8%	<0.001
TALD	4967, 21.1%	2024, 25.6%	2943, 18.8%	<0.001
Associated with Hotels and other hospitality facilities	2453, 10.4%	947, 12.0%	1506, 9.6%	<0.001
in foreigner travelers to Italy, total	2188, 9.3%	864, 10.9%	1324, 8.4%	<0.001
in Italian travelers, total	2779, 11.8%	1160, 14.7%	1619, 10.3%	<0.001
in Italian travelers, abroad	257, 1.1%	111, 1.4%	146, 0.9%	0.001

* calculated for the time period 2007–2019, i.e., 21,158 total cases.

**Table 2:** Incidence of Legionnaires Disease (LD) in Italy (2004–2019). Crude incidence rates (CIR) and corresponding 95% confidence intervals (95%CI) were estimated assuming the total Italian population as the reference. Age-adjusted Incidence Rates (AIR) were calculated by means of the corresponding year census. Healthcare-associated cases of LD (HALD) were reported as percent value over the total of incident cases.

Year	Incident Cases (No./TOTAL, %)	CIR (/100,000 People) [95%CI]	AIR (/100,000 People) [95%CI]	HALD Cases (No., %) [95%CI]
2019	3196 (13.6%)	5.343 (5.131; 5.555)	4.669 (4.251; 5.088)	121, 4.5% (4.3; 4.7)
2018	2960 (12.6%)	4.938 (4.742; 5.135)	4.368 (3.965; 4.770)	100, 4.0% (3.5; 4.5)
2017	2014 (8.6%)	3.353 (3.182; 3.542)	2.988 (2.644; 3.332)	124, 7.9% (7.2; 8.5)
2016	1710 (7.3%)	2.842 (2.705; 2.979)	2.558 (2.260; 2.916)	86, 5.9% (5.4; 6.4)
2015	1569 (6.7%)	2.602 (2.458; 2.746)	2.378 (2.061; 2.696)	88, 6.6% (5.5; 7.8)
2014	1497 (6.4%)	2.481 (2.345; 2.617)	2.305 (1.979; 2.631)	62, 5.0% (4.7; 5.3)
2013	1347 (5.7%)	2.235 (2.106; 2.363)	2.095 (1.809; 2.380)	62, 5.5% (5.2; 5.8)
2012	1350 (5.7%)	2.246 (2.111; 2.381)	2.115 (1.830; 2.401)	72, 6.7% (5.6; 7.8)
2011	1008 (4.3%)	1.681 (1.561; 1.802)	1.593 (1.299; 1.887)	65, 6.5% (6.0; 6.9)
2010	1234 (5.2%)	2.067 (1.939; 2.196)	1.981 (1.732; 2.230)	65, 5.3% (5.0; 5.5)
2009	1200 (5.1%)	2.020 (1.885; 2.154)	1.956 (1.709; 2.203)	110, 9.1% (8.6; 9.6)
2008	1189 (5.1%)	2.015 (1.901; 2.129)	1.969 (1.721; 2.217)	85, 7.1% (6.6; 7.6)
2007	862 (3.7%)	1.473 (1.373; 1.574)	1.441 (1.207; 1.675)	89, 9.3% (8.6; 10.1)
2006	923 (3.9 %)	1.583 (1.478; 1.698)	1.572 (1.293; 1.851)	91, 10.0% (9.2; 10.9)
2005	869 (3.7%)	1.497 (1.405; 1.589)	1.491 (1.233; 1.748)	78, 9.0% (8.5; 9.4)
2004	604 (2.6%)	1.048 (0.958; 1.139)	1.053 (0.869; 1.237)	96, 15.9% (13.5; 18.3)
TOTAL	23,532, 100%	2.464 (1.880; 3.048)	2.285 (1.798; 2.772)	1394, 5.9% (4.5; 7.3))

**Table 3:** Incidence of Legionnaires’ Disease (LD) in Italy (2004–2019) by region. Crude incidence rates (CIR) and corresponding 95% confidence intervals (95%CI) were estimated assuming the regional Italian population as the reference. HALD Risk Ratio was calculated as the ratio between the share of healthcare-associated LD cases (HALD) on the whole of cases reported at national level (No. 1394) and the share of all cases occurring in the same region compared to all notified cases (No. 23,532).

	No./23,532, %	Year Incident Cases No., Average (95%CI)	CIR (/100,000) Average (95%CI)	HALD No./All Cases from the Index Region, % (95%CI)	HALD Risk Ratio (95%CI)
Region					
Abruzzo	268, 1.1%	14.9 (8.4; 21.4)	1.273 (1.146; 1.400)	6, 1.2% (0.9; 1.5)	0.475 (0.345; 0.605)
Basilicata	121, 0.5%	7.3 (5.4; 9.1)	1.313 (1.190; 1.437)	25, 16.2% (13.1; 19.4)	0.472 (0.319; 0.623)
Calabria	82, 0.4%	4.7 (2.9; 6.6)	0.263 (0.218; 0.308)	4, 9.7% (4.3; 15.1)	0.100 (0.061; 0.138)
Campania	1066, 4.5%	59.7 (43.6; 75.7)	1.151 (1.120; 1.183)	46, 5.9% (5.5; 6.2)	0.439 (0.374; 0.504)
Emilia Romagna	3035, 12.9%	172.6 (104.0; 241.2)	4.317 (4.200; 4.433)	187, 7.2% (7.1; 7.3)	1.608 (1.322; 1.894)
Friuli Venezia Giulia	427, 1.8%	24.2 (15.9; 32.6)	2.195 (2.058; 2.333)	19, 4.6% (3.9; 5.4)	0.845 (0.741; 0.949)
Lazio	2175, 9.2%	172.6 (104.0; 241.2)	2.422 (2.378; 2.466)	126, 5.2% (5.1; 5.3)	1.019 (0.925; 1.114)
Liguria	703, 3.0%	40.6 (27.5; 53.7)	2.808 (2.674; 2.943)	43, 6.3% (6.0; 6.6)	1.123 (0.963; 1.284)
Lombardy	7829, 33.3%	464.5 (361.0; 568.0)	4.999 (4.952; 5.046)	487, 6.3% (6.2; 6.4)	2.126 (2.056; 2.197)
Marche	527, 2.2%	29.6 (19.3; 39.8)	2.151 (2.033; 2.268)	25, 4.9% (4.4; 5.3)	0.826 (0.684; 0.967)
Molise	10, 0.1%	0.6 (0.1; 1.0)	0.202 (0.001; 0.409)	1, 7.8% (0.1; 23.5)	0.043 (0.001; 0.085)
AP of Trento	666, 2.8%	37.6 (29.8; 45.3)	7.919 (7.749; 8.088)	125, 18.0% (17.4; 18.6)	2.855 (2.479; 3.232)
AP of Bolzano	274, 1.2%	15.3 (10.2; 20.4)	3.338 (3.106; 3.570)	11, 4.7% (3.9; 5.6)	1.201 (0.844; 1.557)
Piemonte	1549, 6.6%	95.4 (79.6; 111.1)	2.228 (2.189; 2.267)	172, 9.1% (8.6; 9.5)	0.947 (0.811; 1.084)
Puglia	433, 1.8%	25.3 (16.6; 34.0)	0.670 (0.626; 0.714)	30, 6.1% (5.7; 6.5)	0.264 (0.223; 0.305)
Sardinia	126, 0.5%	7.4 (4.3; 10.5)	0.480 (0.407; 0.553)	4, 3.2% (1.7; 4.7)	0.190 (0.134; 0.247)
Sicily	180, 0.8%	10.3 (7.1; 13.4)	0.225 (0.206; 0.244)	13, 6.3% (4.6; 8.0)	0.093 (0.068; 0.117)
Tuscany	1994, 8.5%	116.6 (90.1; 143.1)	3.374 (3.310; 3.458)	135, 6.9% (6.8; 7.1)	1.393 (1.293; 1.493)
Umbria	404, 1.7%	22.9 (17.6; 28.1)	2.876 (2.771; 2.981)	20, 6.9% (5.8; 8.0)	1.141 (0.953; 1.329)
Aosta Valley	72, 0.3%	4.6 (3.5; 5.6)	3.571 (3.182; 3.961)	6, 5.4% (0.4; 10.3)	1.635 (1.302; 1.967)
Veneto	1613, 6.9%	95.3 (72.6; 117.9)	2.078 (2.031; 2.125)	61, 3.9% (3.8; 4.1)	0.457 (0.767; 0.927)
Area					
Northwestern Italy	10,153, 43.2%	605.1 (475.0; 735.2)	3.527 (2.895; 4.158)	708, 7.0% (7.0; 7.1)	1.697 (1.500; 1.894)
Northeastern Italy	6015, 25.6%	345.0 (237.3; 452.7)	2.572 (1.885; 3.258)	403, 7.5% (7.4; 7.6)	1.244 (1.055; 1.432)
Central Italy	5368, 22.8%	313.0 (239.2; 386.8)	2.190 (1.757; 2.622)	312, 6.0% (5.9; 6.1)	1.068 (0.951; 1.185)
Southern Italy	1712, 7.3%	97.6 (71.4; 123.7)	0.671 (0.499; 0.843)	106, 7.2% (6.9; 7.5)	0.322 (0.271; 0.374)
Islands	306, 1.3%	17.7 (12.3; 23.0)	0.243 (0.172; 0.314)	17, 5.5% (4.6; 6.4)	0.117 (0.091; 0.143)

**Table 4:** Care fatality Ratio for Legionnaire’s Disease (LD) between 2004 and 2019, and in the timeframes 2004–2011, 2012–2019, broken down as community cases and cases in healthcare/residential homes.

	Total Cases		Community Cases		Healthcare Related Cases		*p* Value (Chi Squared Test)
	Cases (No.)	Deaths (No., %)	Cases (No.)	Deaths (No., %)	Cases (No.)	Deaths (No., %)	
2004–2019	23,554	1223, 5.2%	21,637	1002, 4.6%	1917	221, 11.5%	<0.001
2004–2011	7904	461, 5.8%	7083	352, 5.0%	821	109, 13.3%	<0.001
2012–2019	15,650	762, 4.9%	14,556	650, 4.5%	1094	112, 10.2%	<0.001

**Table 5:** Occurrence of Legionnaire’s Disease (LD) in Italy 2004–2019 with calculation of corresponding risk ratios and 95% confidence intervals.

		Risk Ratio	95% Confidence Intervals
Diagnosis by timeframe	2004–2011	1.000	Reference
	2012–2019	1.936	1.884; 1.989
Gender	Female	1.000	Reference
	Male	2.522	2.453; 2.594
Age group	<40	1.000	Reference
	40 to 49	4.320	4.051; 4.606
	50 to 59	6.259	5.894; 6.647
	60 to 69	8.145	7.676; 8.462
	70 to 79	9.610	9.058; 10.197
	80 or greater	9.447	9.361; 10.565
Geographic Origin	Northwestern Italy	1.000	Reference
	Northeastern Italy	0.819	0.793; 0.845
	Central Italy	0.643	0.622; 0.665
	Southern Italy	0.209	0.199; 0.220
	Islands	0.071	0.064; 0.080
Deaths	Community cases	1.000	Reference
	Healthcare/Residential homes	2.489	2.169; 2.858
	2004–2011	1.000	Reference
	2012–2019	0.835	0.746; 0.934

**Table 6:** Correlation of outcome variables total incident cases of Legionnaires’ Disease (model 1) and healthcare-related cases (model 2), with average temperatures, daily precipitation rates, share of total population aged 50 years or more, and income (both for renters and for general population).

	Total Incident Cases (Model 1)		Healthcare-Related Cases (Model 2)	
	r	*p* Value	r	*p* Value
Air Temperature	−0.203 (−0.429; 0.047)	0.110	−0.309 (−0.518; −0.067)	0.014
Daily Precipitation	0.167 (−0.085; 0.398)	0.192	−0.004 (−0.251; 0.244)	0.978
% of Population aged 50 years or more	0.405 (0.175; 0.594)	<0.001	−0.193 (−0.420; 0.058)	0.131
Income (renters)	0.695 (0.540; 0.804)	<0.001	−0.416 (−0.602; −0.188)	<0.001
Income (general population)	0.643 (0.470; 0.768)	<0.001	−0.259 (−0.476; 0.012)	0.041

**Table 7:** Incidence rate ratios (IRR) for Legionnaire’s Disease (LD) cases in Italy (2004–2019) by meteorological factors, and main characteristics of the index areas. IRR were calculated by means of a Poisson logistic regression assuming as outcome variable the annual incident cases of LD, both in total (total incident cases) and healthcare-related ones.

	Total Incident Cases (Model 1)		Healthcare-Related Cases (Model 2)	
	IRR	95%CI	IRR	95%CI
Air Temperature (+1.0 °C)	0.807	0.744; 0.874	0.884	0.783; 0.999
Daily Precipitation (+1.0 kg/m^2^)	1.035	0.988; 1.085	1.046	0.974; 1.124
% of Population aged 50 yrs or more	2.979	0.012; 720.819	0.727	0.001; 441.993
Income (general, +1000€/yr)	1.019	0.957; 1.087	0.998	0.916; 1.088
Income (renter, +1000€/yr)	1.238	1.134; 1.351	0.888	0.816; 0.966

## Data Availability

The data presented in this study are available on request from the corresponding author.

## Appendix A

**Table A1:** Cases of Legionnaires’ Disease reported among Italian travelers. Crude incidence rates were calculated assuming as a reference the total nights abroad spent by Italian Travelers according to the Italian Institute for Statistics (ISTAT).

Year	Incident Cases (No./257; %)	Incident Cases (per 1,000,000 Nights Abroad)
2019	26, 10.3%	0.202
2018	33, 12.8%	0.266
2017	16, 6.2%	0.030
2016	12, 4.8%	0.146
2015	23, 9.0%	0.239
2014	13, 5.1%	0.125
2013	12, 4.5%	0.166
2012	11, 4.3%	0.128
2011	9, 3.5%	0.097
2010	13, 5.0%	0.117
2009	18, 6.9%	0.140
2008	13, 5.2%	0.102
2007	17, 6.5%	0.130
2006	19, 7.3%	0.153
2005	11, 4.3%	0.092
2004	11, 4.1%	0.093
AVERAGE (95% Confidence interval)	16.0 (13.1; 24.0)	0.148 (0.110; 0.185)

**Table A2:** Daily consumption of fluoroquinolones according to the available annual reports of Italian Medicines Agency (i.e., Agenzia Italiana del Farmaco, AIFA in Italian) (DDD = defined daily doses; i.e., The assumed average maintenance dose per day for a drug used for its main indication in adults.

Year	Geographic Area	Fluoroquinolones (DDD, % on Total Doses)	Total (DDD)
2007	National level	3.5, 11.7%	30.6
	Northern Italy	2.8, 12.3%	22.5
	Central Italy	3.8, 13.0%	29.2
	Southern Italy	4.1, 10.3%	40.1
2017	National level	2.7, 13.7%	19.7
	Northern Italy	2.0, 12.8%	15.6
	Central Italy	2.9, 14.0%	20.7
	Southern Italy	3.6, 14.6%	24.9
2018	National level	2.6, 16.1%	16.1
	Northern Italy	1.9, 15.0%	12.7
	Central Italy	2.8, 16.6%	16.9
	Southern Italy	3.5, 17.2%	20.4
2019	National level	1.9, 12.2%	15.6
	Northern Italy	1.3, 10.4%	12.4
	Central Italy	2.0, 11.9%	16.8
	Southern Italy	2.6, 13.3%	19.6
